# Feasibility of a pragmatic clinical trial model in Lewy body disease and its effects on recruitment, retention, and diversity of study participants

**DOI:** 10.1002/alz70858_098408

**Published:** 2025-12-24

**Authors:** Sarah Horn, Chen‐Pin Wang, Pablo Coss, Okeanis E. Vaou, Leila Saadatpour, Carolyn Paiz, Diana Solis, Jessica Grimaldo, Sudha Seshadri

**Affiliations:** ^1^ University of Texas Health Science Center at San Antonio, San Antonio, TX, USA; ^2^ Glenn Biggs Institute for Alzheimer's & Neurodegenerative Diseases, University of Texas Health Science Center, San Antonio, TX, USA; ^3^ Glenn Biggs Institute for Alzheimer's and Neurodegenerative Diseases, University of Texas Health Science Center, San Antonio, TX, USA

## Abstract

**Background:**

Both quetiapine and pimavanserin are commonly used in clinical practice for psychosis (hallucinations and/or delusions) in Parkinson's disease (PD) and dementia with Lewy bodies (DLB) ‐ collectively referred to as Lewy body disease (LBD). Clinical trials to establish efficacy have been small, with mixed results, indicating the classic clinical trial model may be ill‐equipped to capture the real‐world benefits seen in routine clinical practice. Historically, the rigorous nature of traditional, randomized, double‐blind, placebo‐controlled clinical trials evaluating treatments for dementia and neuropsychiatric symptoms of PD has been fraught with recruitment and retention issues. Furthermore, there has been a lack of racial and ethnic diversity among participants in published clinical trials in these populations, thereby limiting their power and generalizability to the general population. Notably, less than 1% of participants in published trials pertaining to neuropsychiatric symptoms in PD are Hispanic.

**Method:**

An investigator‐initiated, local, pragmatic, randomized, unblinded clinical trial comparing quetiapine and pimavanserin for the treatment of LBD psychosis is ongoing in San Antonio, Texas, which is a majority‐Hispanic city. The medication chosen via randomization is prescribed and managed per routine care. Baseline and follow‐up trial visits are performed during routine appointments, intertwined with clinical care. The primary outcome measure of the trial is change in the sum of the Neuropsychiatric Inventory Questionnaire (NPI‐Q) hallucination and delusion items, from baseline to 6 months. Secondary outcome measures include change in NPI‐Q total score, Global Impression of Change, adverse events, medication cost, discontinuation of per‐protocol medication, and changes in anxiety, depression, apathy, or sleep (NPI‐Q sub scores).

**Result:**

Among 49 participants (mean age 75.2, SD 8.3) enrolled into the study to date (24 pimavanserin, 25 quetiapine), 41 (83.7%) have PD and 8 (16.3%) have DLB; 19 (38.8%) are women; 20 (40.8%) identify as Hispanic, reflective of the clinic population; and 2 have been lost to follow‐up (1 death, 1 withdrew after moving).

**Conclusion:**

Pragmatic clinical trials are feasible for patients with psychosis due to LBD. The pragmatic clinical trial model can ease recruitment and retention issues and increase representation of underrepresented minorities to reflect the clinic population.

